# Supplementary figures and images for: Contrasting Patterns of Fungal and Bacterial Endophytes Inhabiting Temperate Tree Leaves in Response to Thinning
Source: J Fungi (Basel). 2024 Jul 5;10(7):470. doi: 10.3390/jof10070470 (PMC11277613; doi:10.3390/jof10070470)

(a)

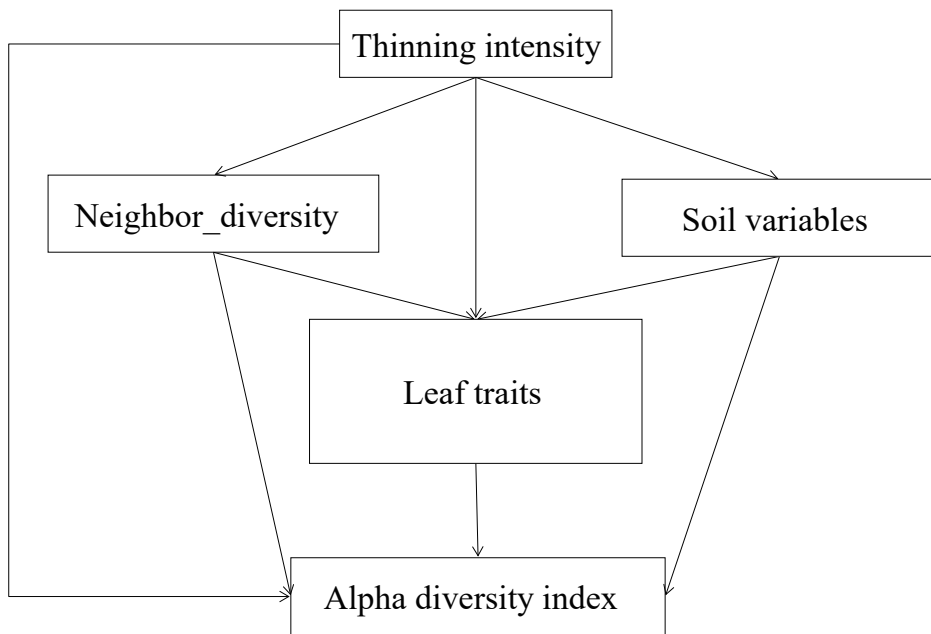

(b)

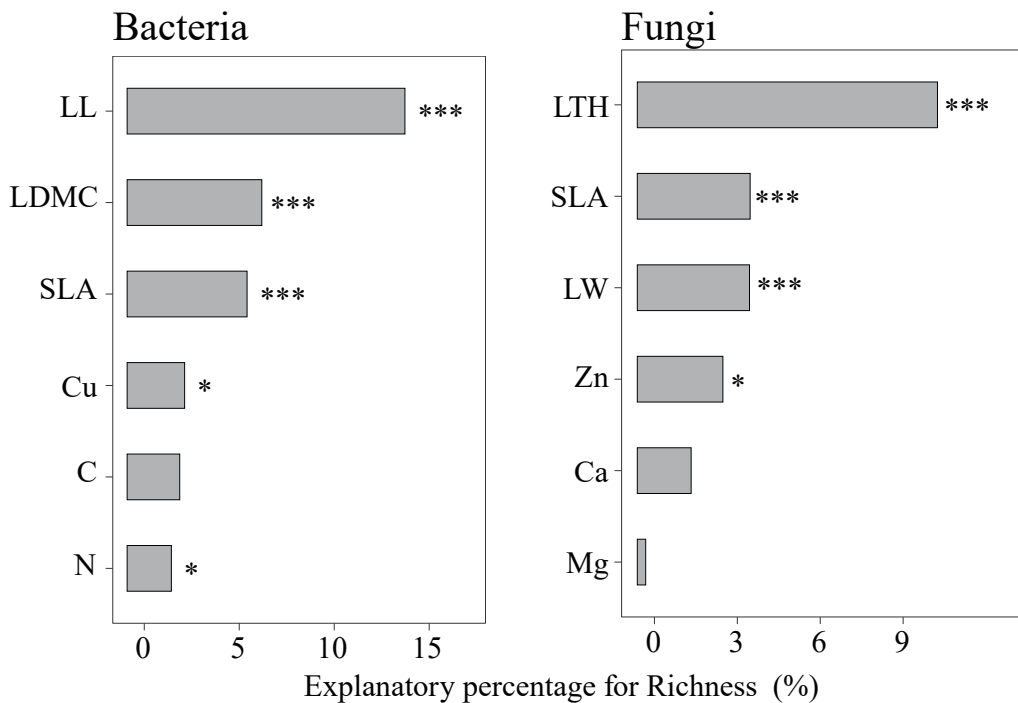

Supplement: Supplementary file 1 [file jof-10-00470-s001.zip › Figure_S1.pdf]

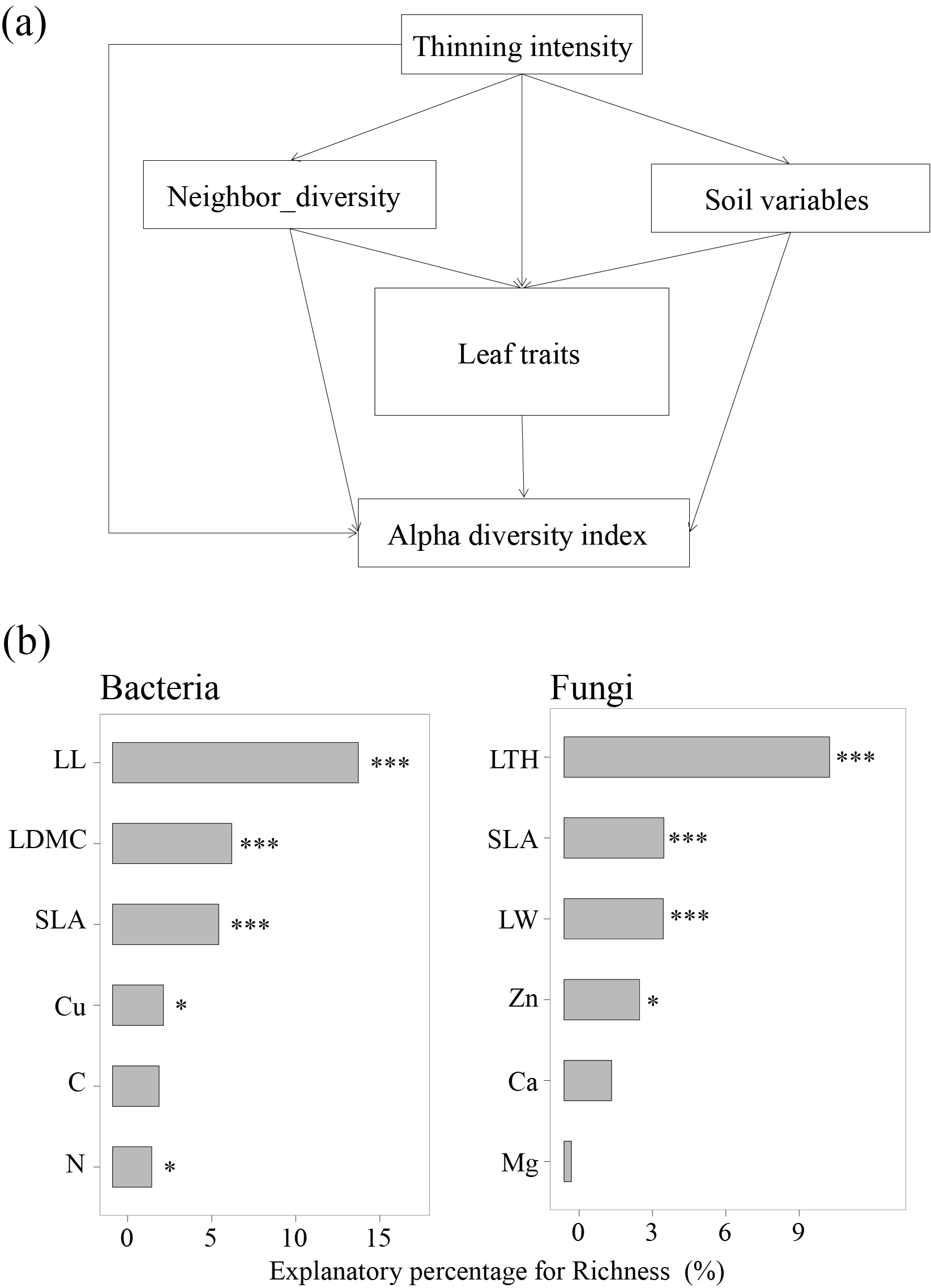

Supplement: Supplementary file 1 [file jof-10-00470-s001.zip › Figure_S1.png]

## Bacteria

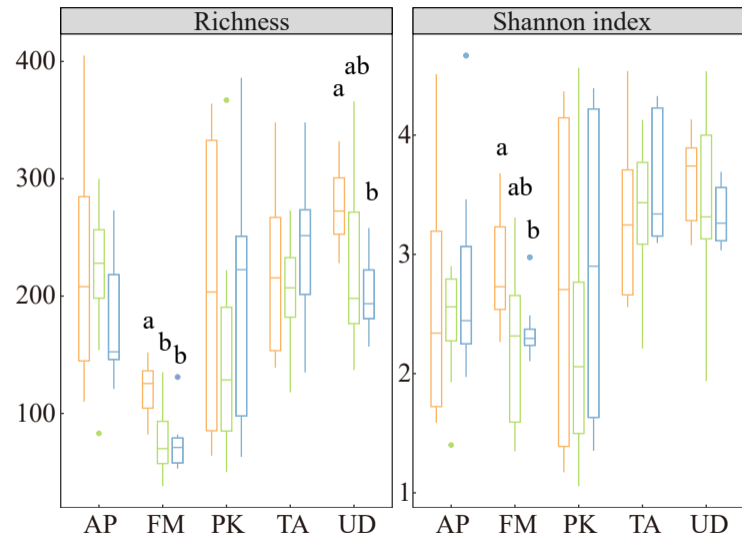

## Fungi

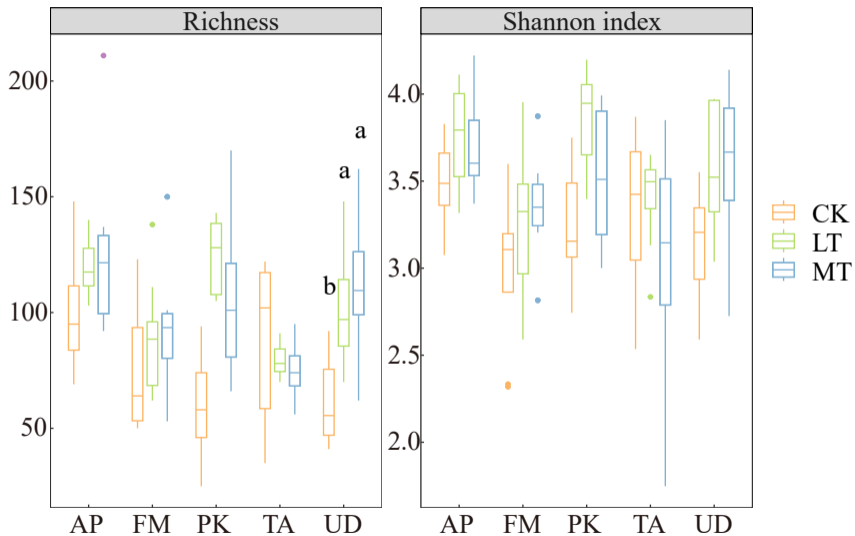

Supplement: Supplementary file 1 [file jof-10-00470-s001.zip › Figure_S2.pdf]

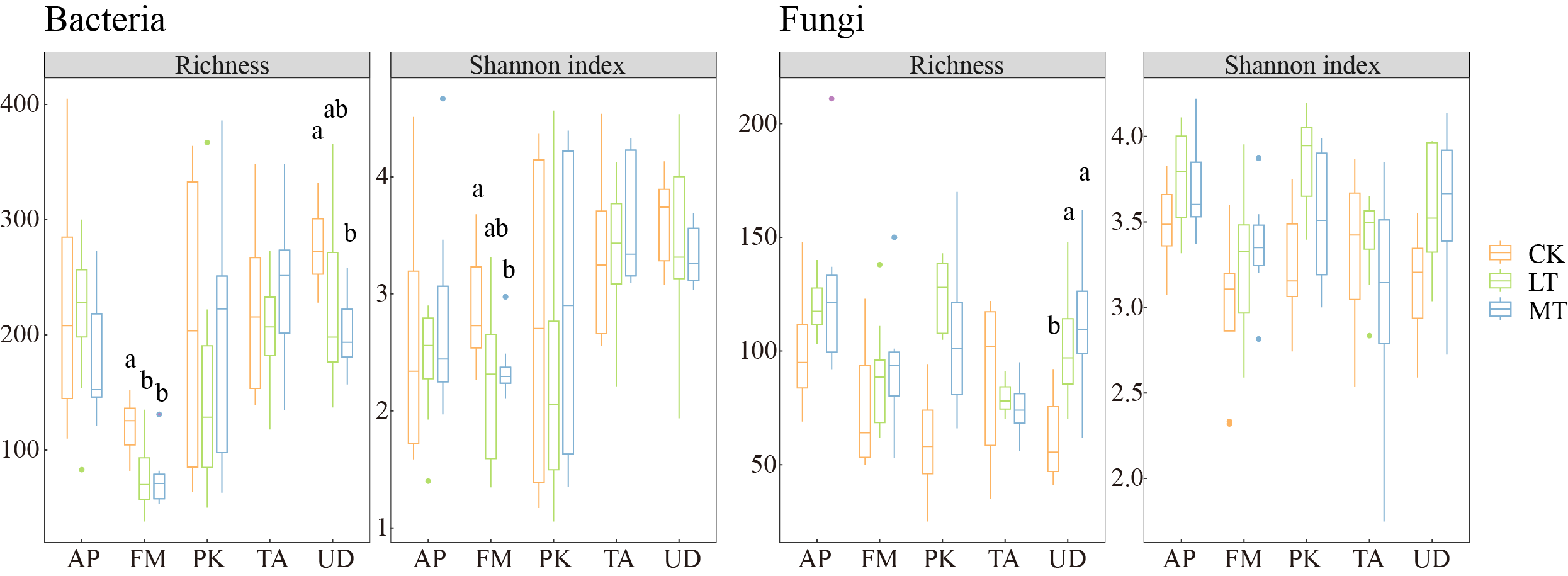

Supplement: Supplementary file 1 [file jof-10-00470-s001.zip › Figure_S2.png]

# Bacteria

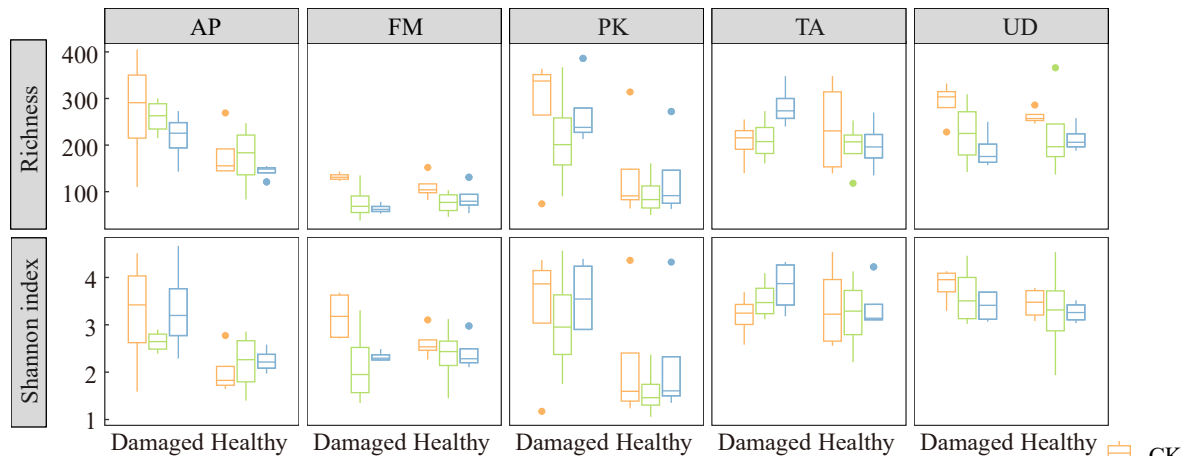

# Fungi

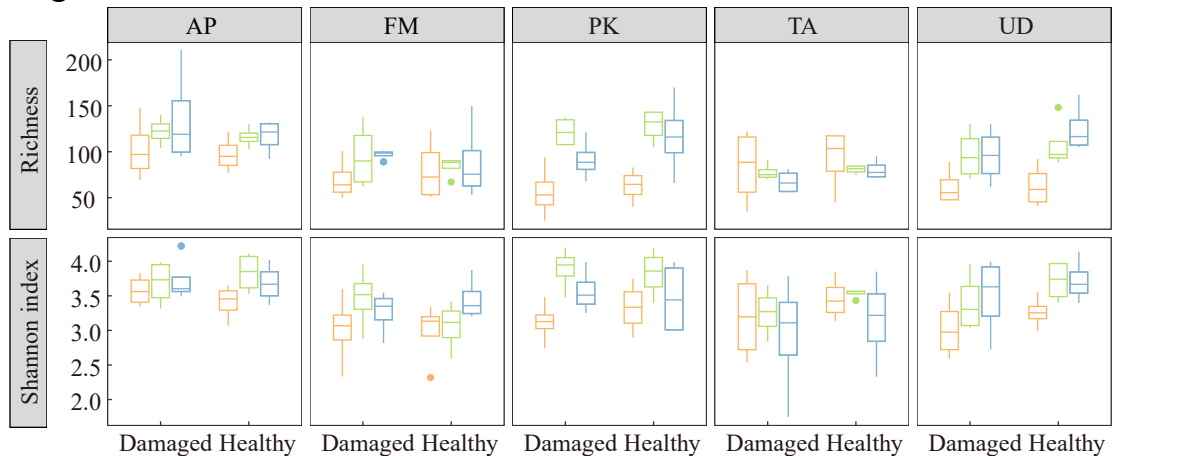

Supplement: Supplementary file 1 [file jof-10-00470-s001.zip › Figure_S3.pdf]

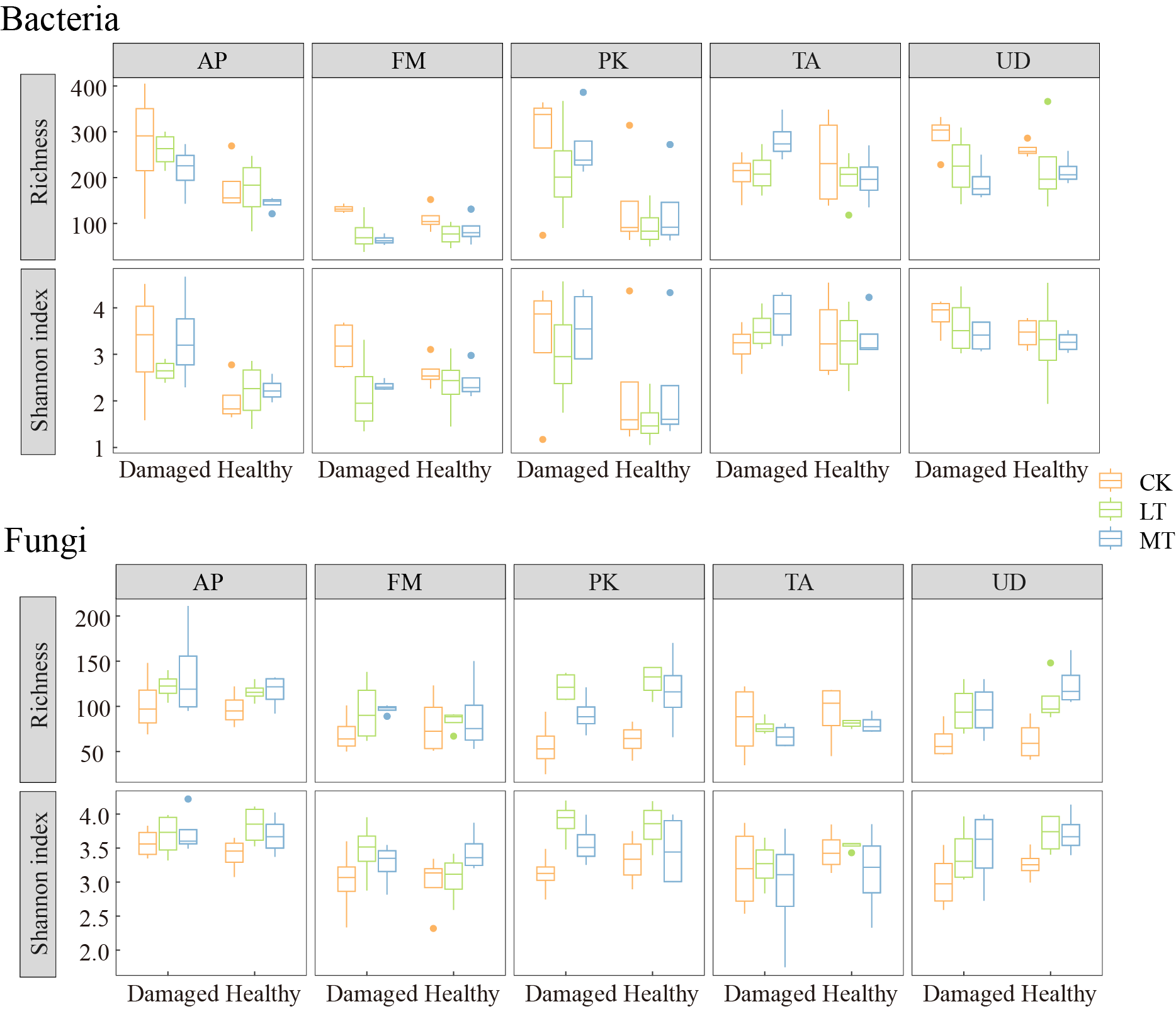

Supplement: Supplementary file 1 [file jof-10-00470-s001.zip › Figure_S3.png]

## Bacteria

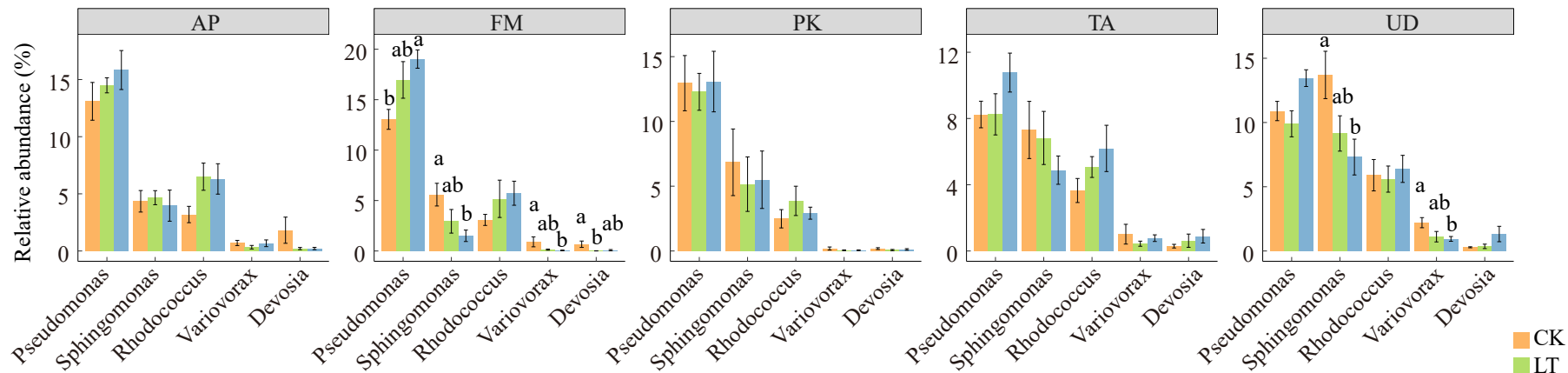

## Fungi

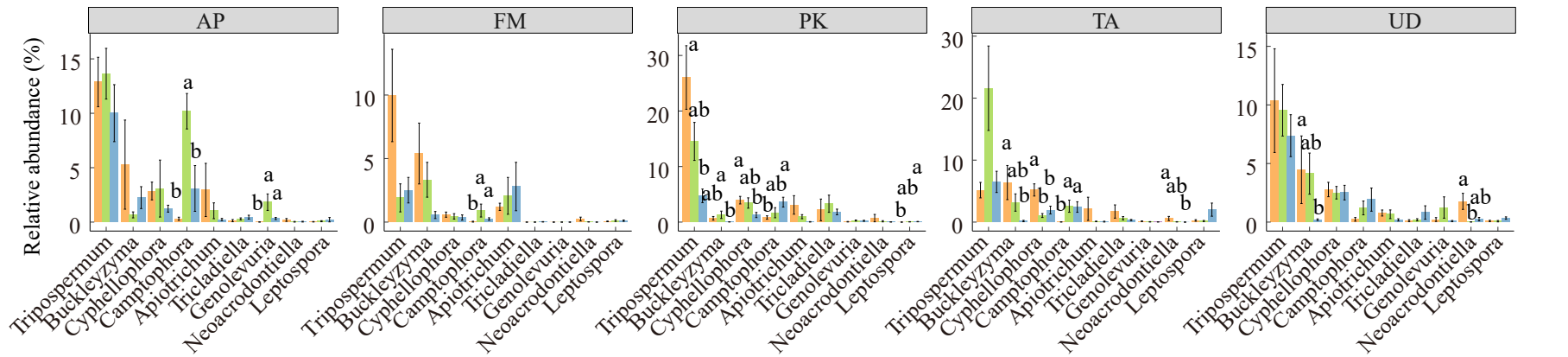

Supplement: Supplementary file 1 [file jof-10-00470-s001.zip › Figure_S4.pdf]

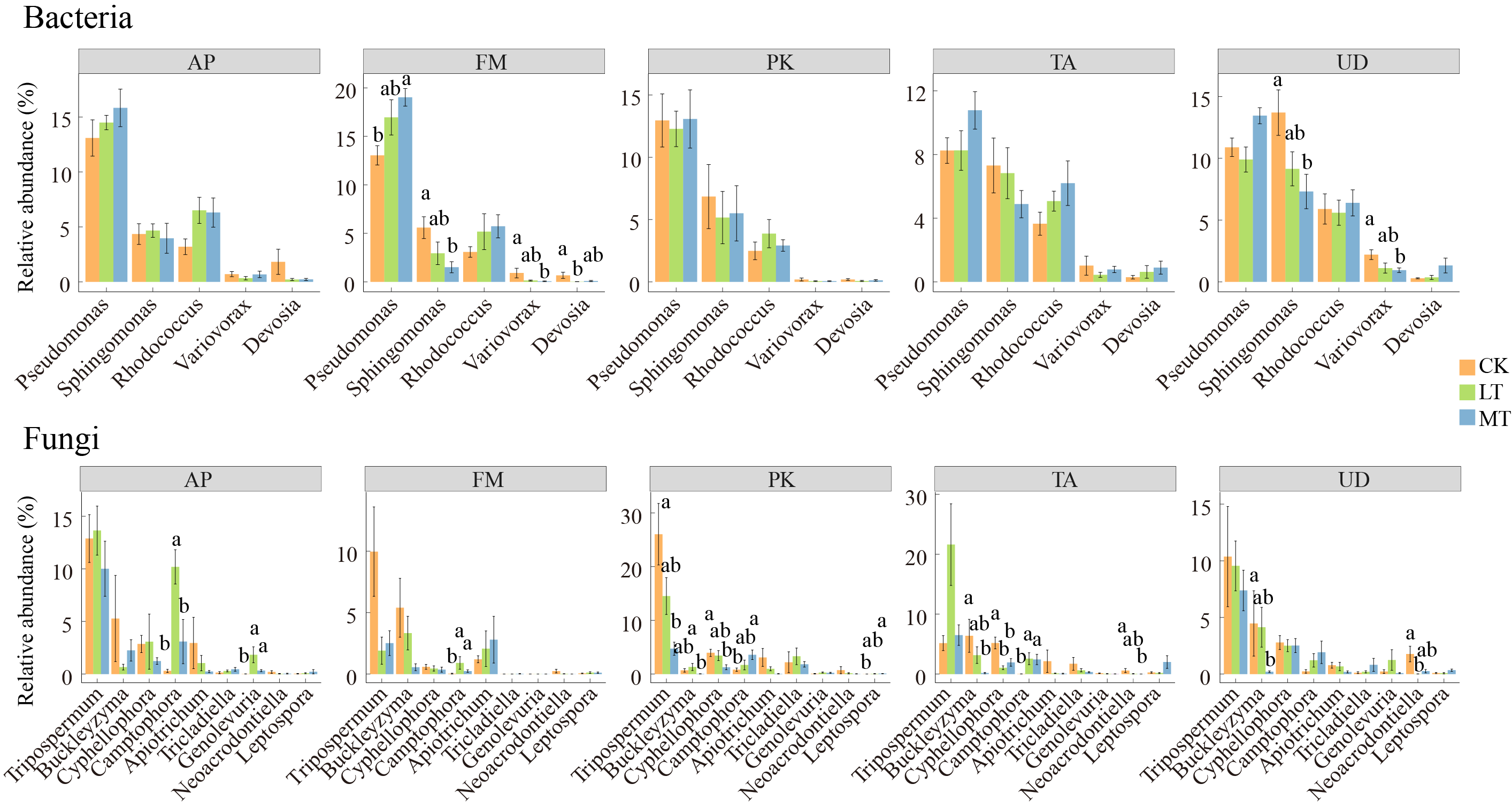

Supplement: Supplementary file 1 [file jof-10-00470-s001.zip › Figure_S4.png]

## Bacteria

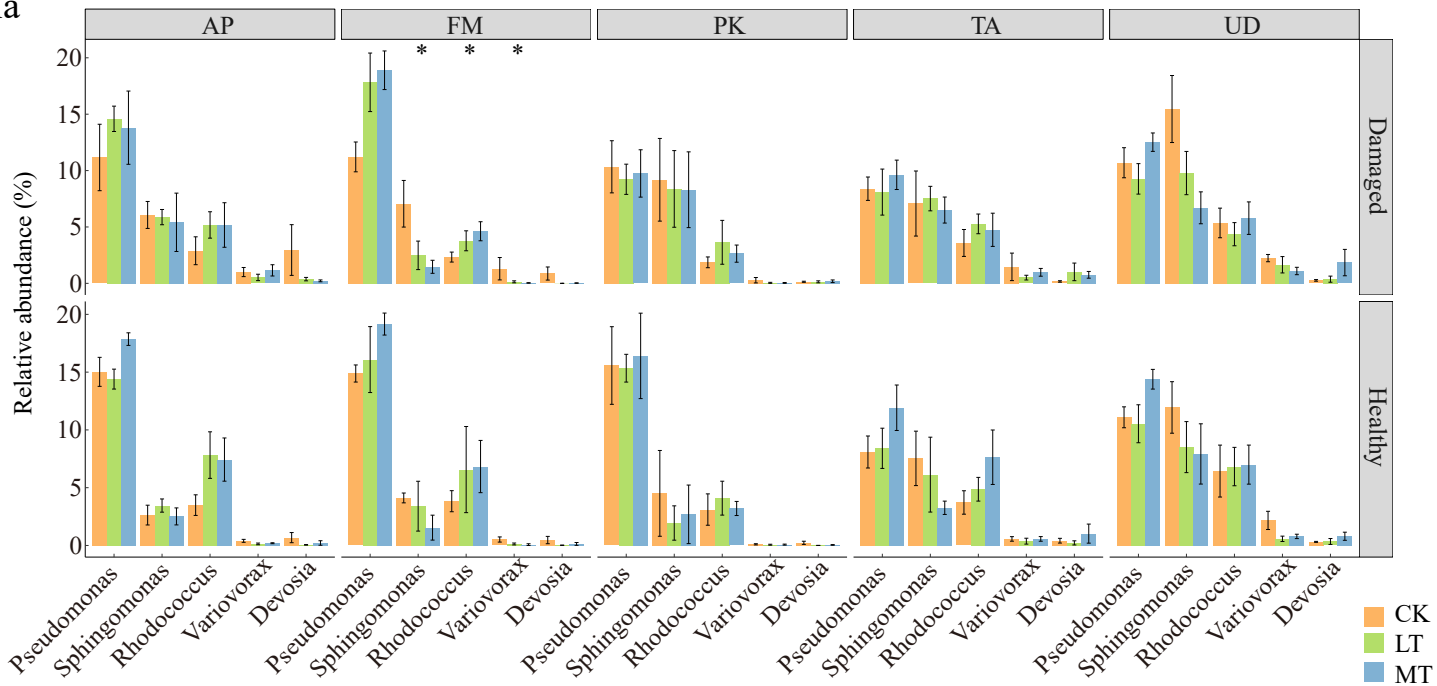

## Fungi

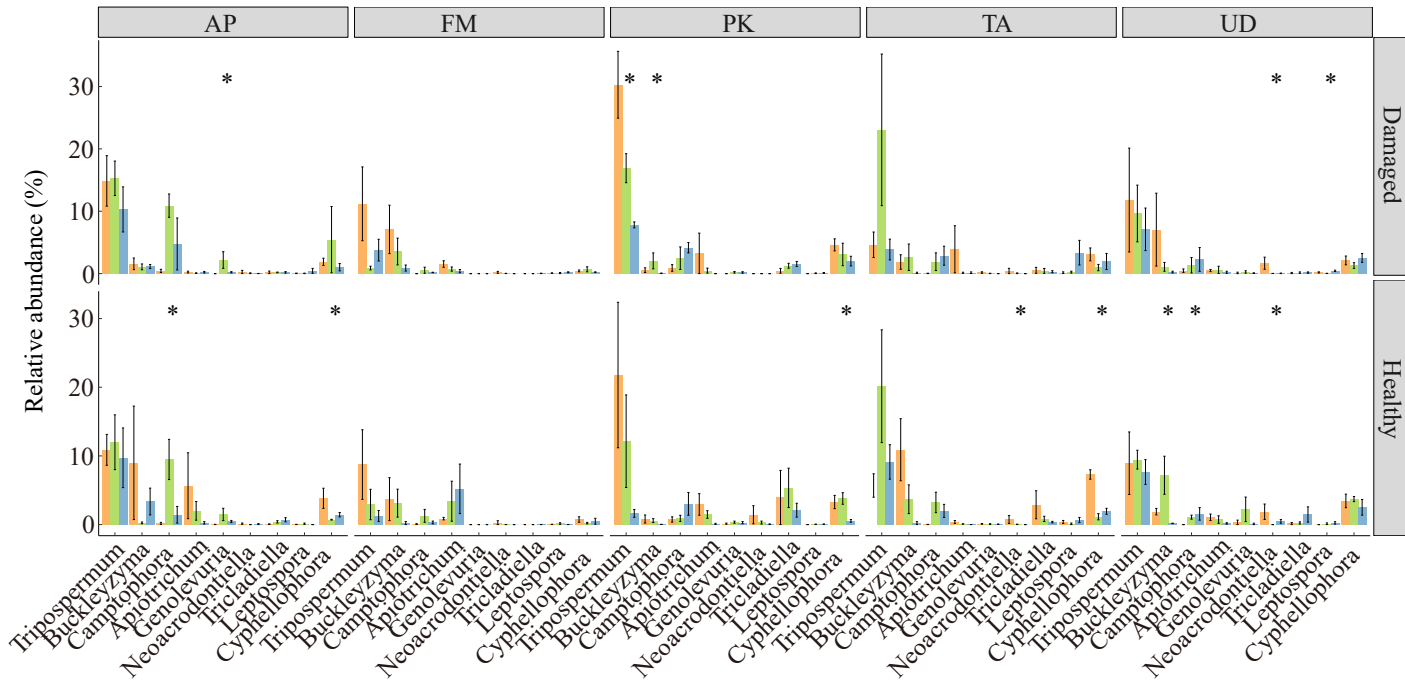

Supplement: Supplementary file 1 [file jof-10-00470-s001.zip › Figure_S5.pdf]

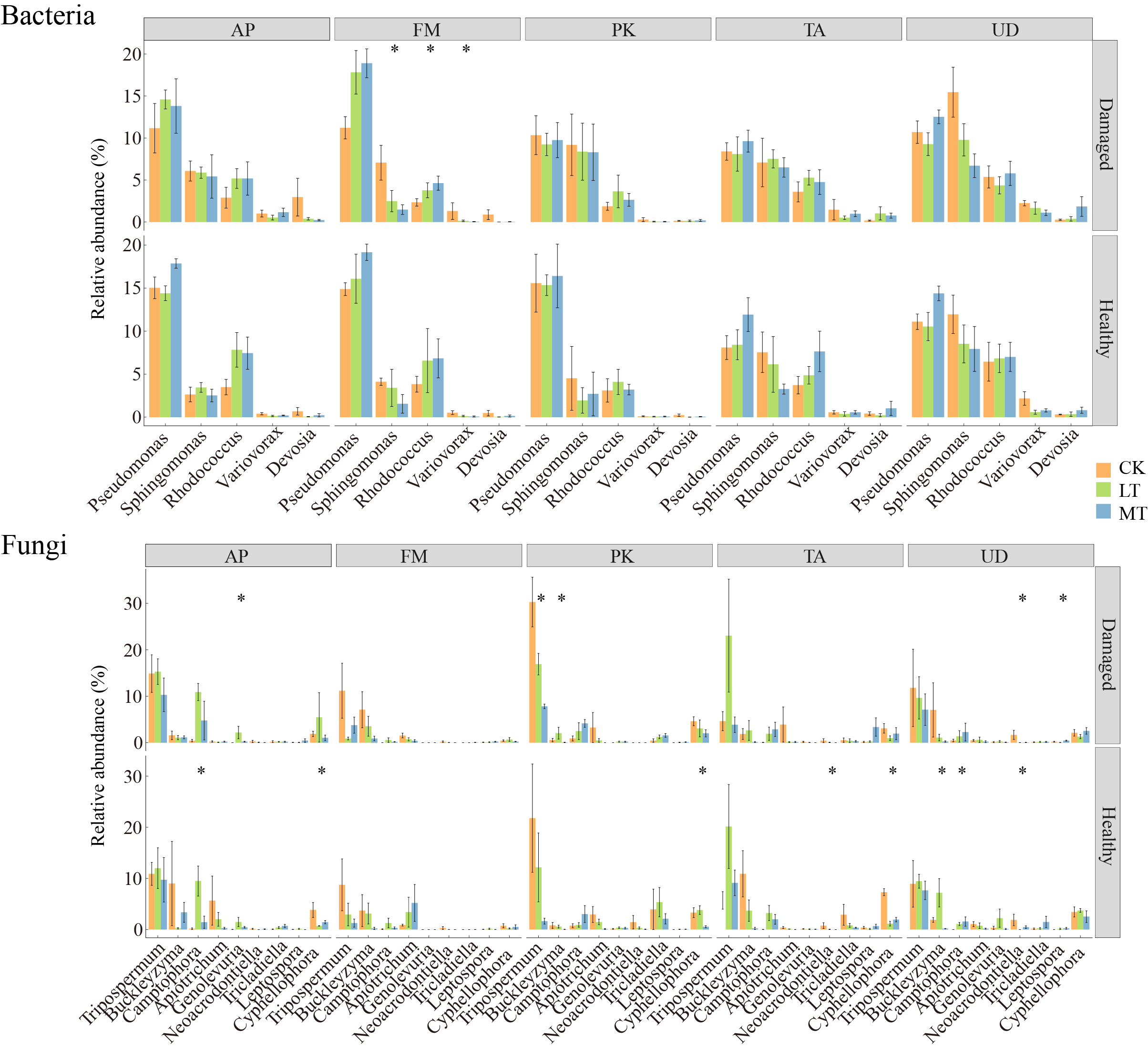

Supplement: Supplementary file 1 [file jof-10-00470-s001.zip › Figure_S5.png]

Bacteria

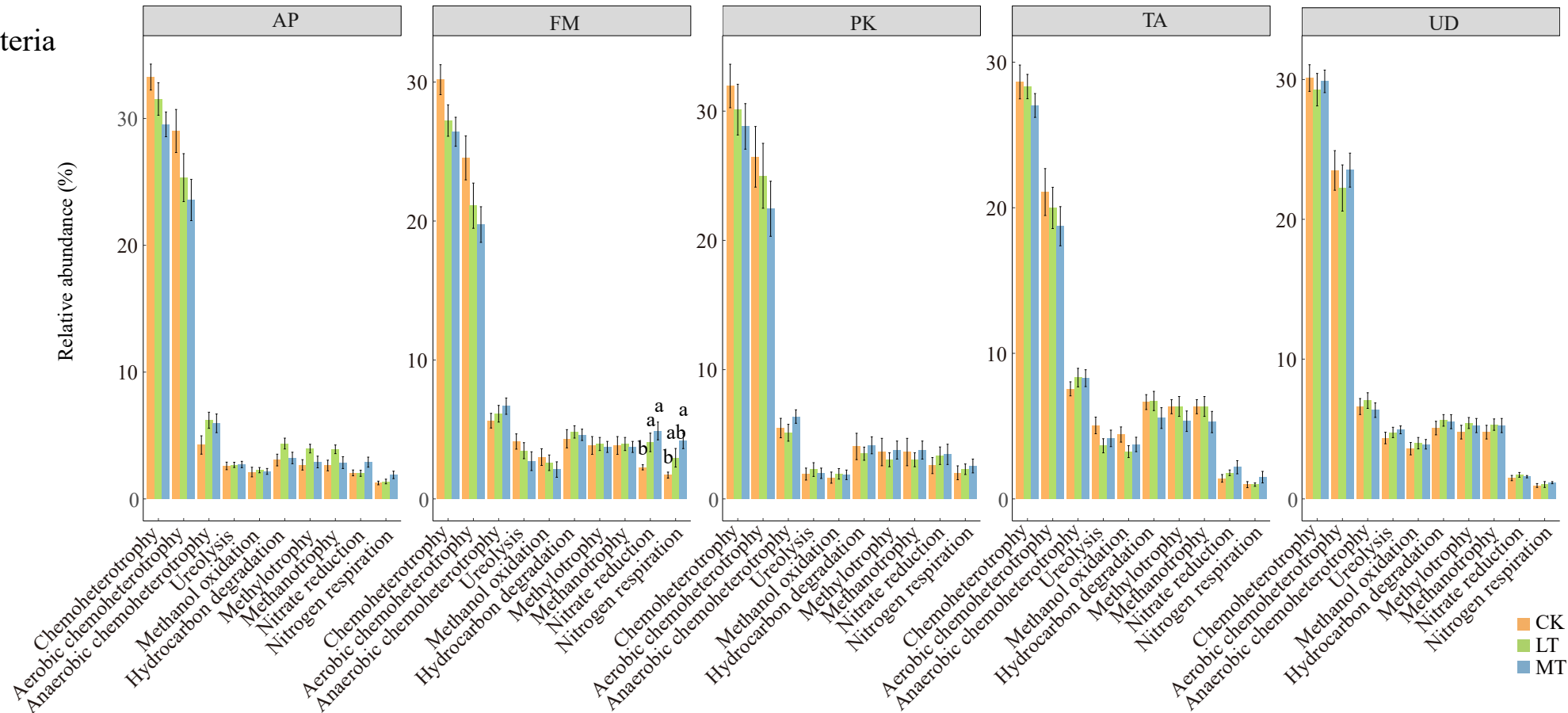

Fungi

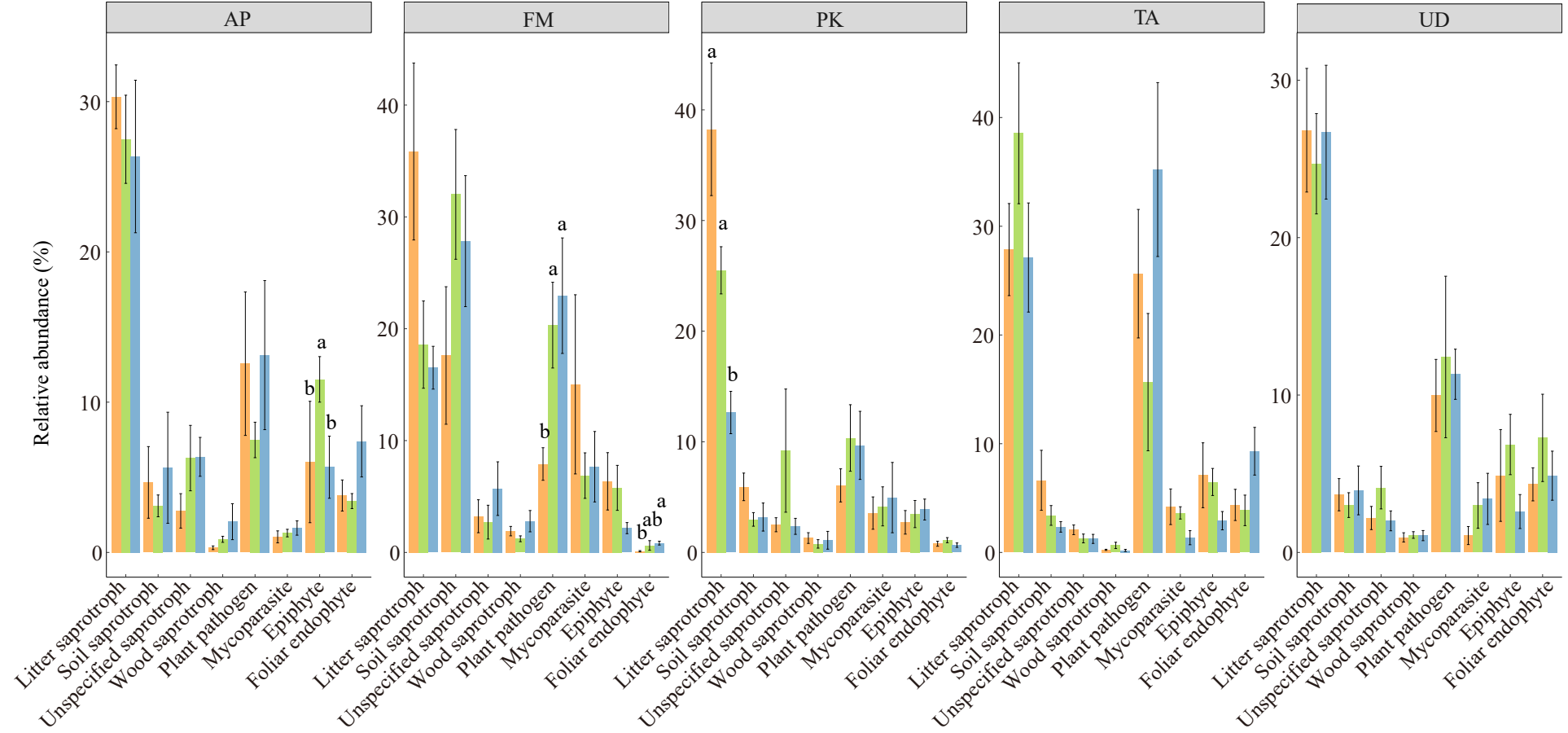

Supplement: Supplementary file 1 [file jof-10-00470-s001.zip › Figure_S6.pdf]

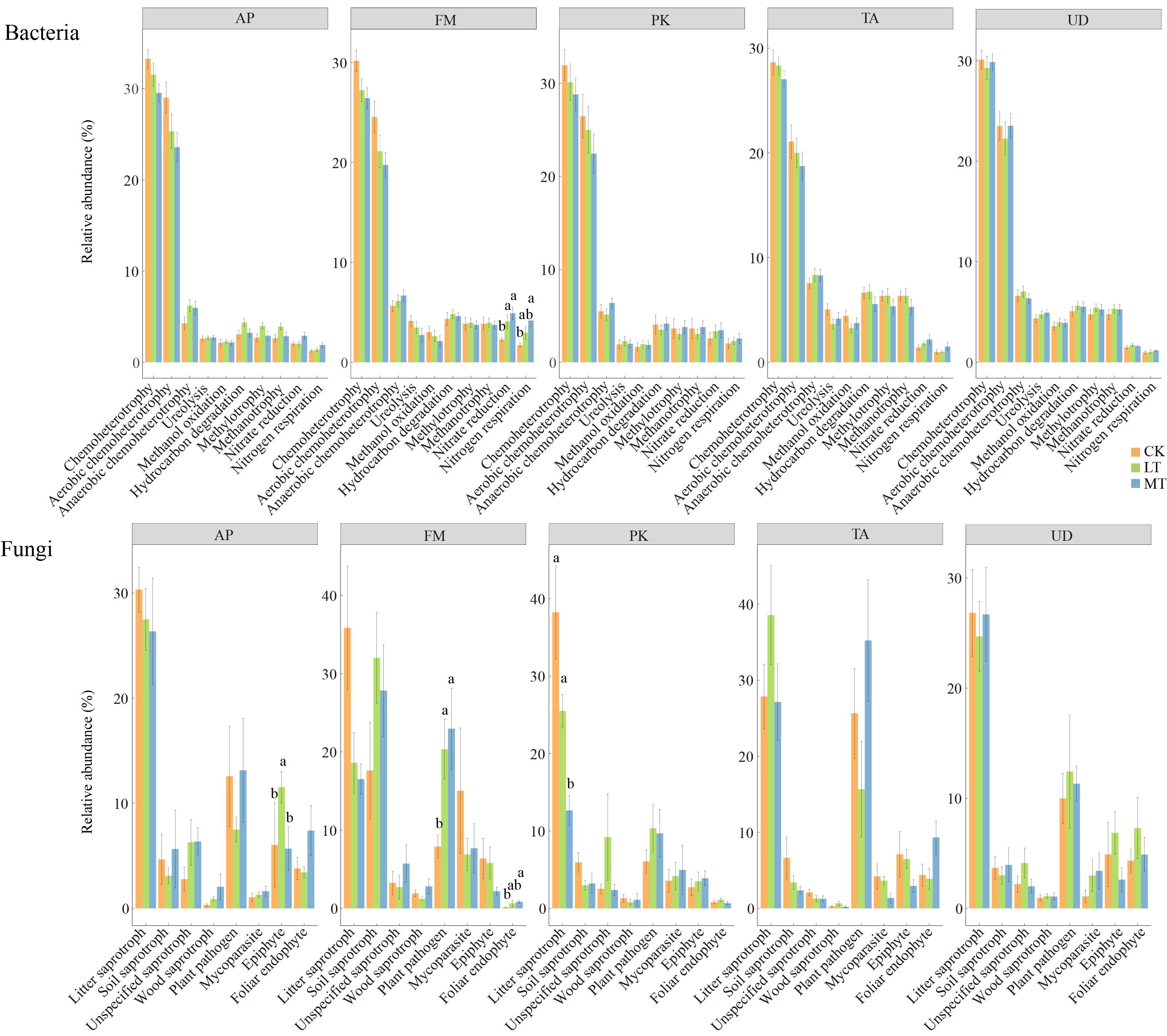

Supplement: Supplementary file 1 [file jof-10-00470-s001.zip › Figure_S6.png]

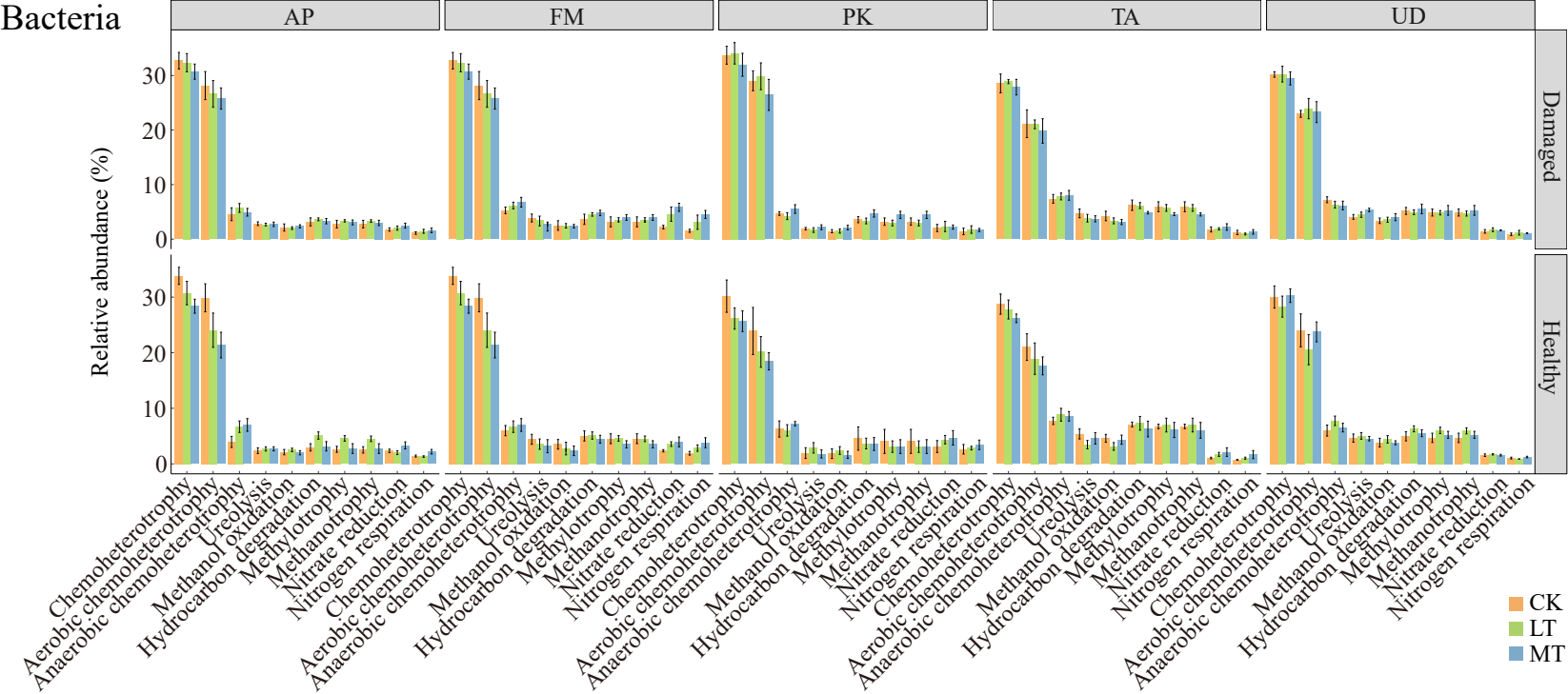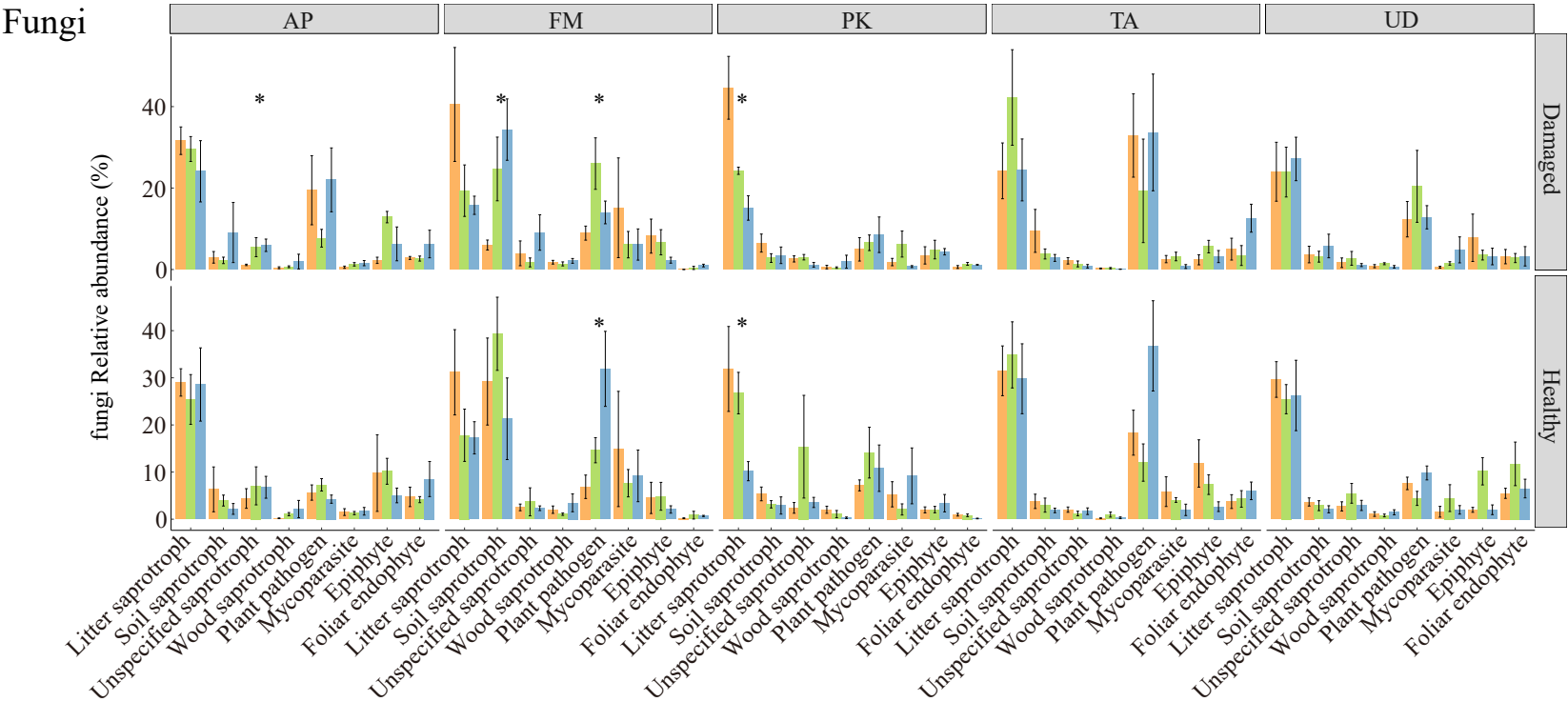

Supplement: Supplementary file 1 [file jof-10-00470-s001.zip › Figure_S7.pdf]

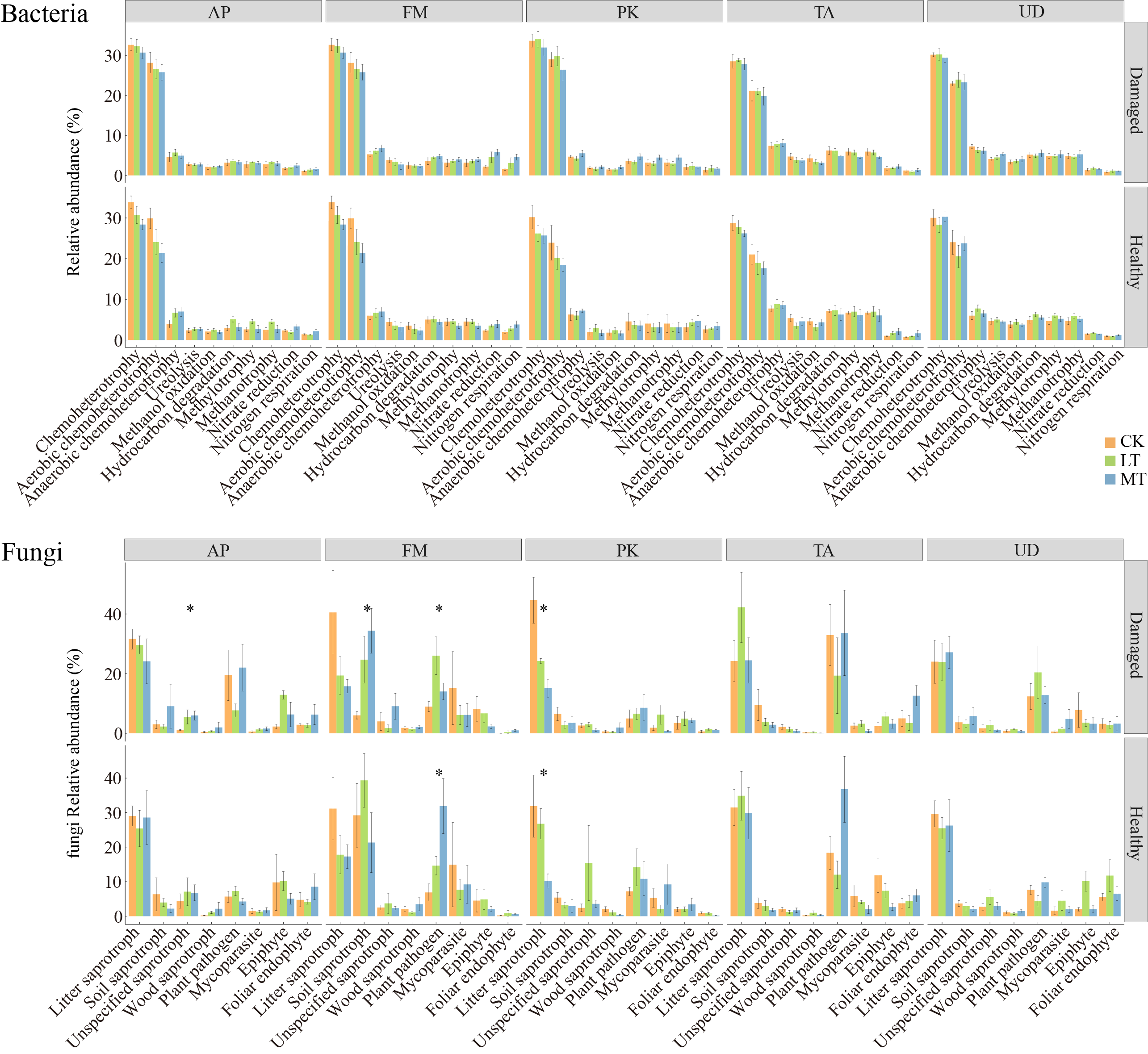

Supplement: Supplementary file 1 [file jof-10-00470-s001.zip › Figure_S7.png]
